# Evaluation of Osteogenic Potential for Rat Adipose-Derived Stem Cells under Xeno-Free Environment

**DOI:** 10.3390/ijms242417532

**Published:** 2023-12-15

**Authors:** Yuzhu Sun, Jun-Ichiro Jo, Yoshiya Hashimoto

**Affiliations:** Department of Biomaterials, Osaka Dental University, 8-1 Kuzuhahanazonocho, Hirakata 573-1121, Osaka, Japan; sun-y@cc.osaka-dent.ac.jp (Y.S.); yoshiya@cc.osaka-dent.ac.jp (Y.H.)

**Keywords:** three-dimensional scaffold, rat adipose-derived stem cells (rADSC), xeno-free culture, osteogenic differentiation, rat congenital cleft-jaw model, bone repair

## Abstract

This study aimed to develop a novel culture method for rat adipose-derived stem cells (rADSC) and evaluate their osteogenic potential. The rADSC cultured in xeno-free culture medium (XF-rADSCs) or conventional culture medium containing fetal bovine serum (FBS-rADSCs) were combined with micropieces of xeno-free recombinant collagen peptide to form 3-dimensional aggregates (XF-rADSC-CellSaic or FBS-rADSC-CellSaic). Both FBS-rADSC and XF-ADSC in CellSaic exhibited multilineage differentiation potential. Compared to FBS-rADSC-CellSaic, XF-rADSC-CellSaic accelerated and promoted osteogenic differentiation in vitro. When transplanted into rat mandibular congenital bone defects, the osteogenically differentiated XF-rADSC-CellSaic induced regeneration of bone tissue with a highly maturated structure compared to FBS-rADSC-CellSaic. In conclusion, XF-rADSC-CellSaic is a feasible 3-dimensional platform for efficient bone formation.

## 1. Introduction

Deficiency of bone tissue in the maxillofacial region caused by congenital abnormalities, inflammation, tumors, and trauma is one of the critical challenges facing the dental field [1,2,3]. Currently, the primary method to treat bone defects involves autologous, allogenic, and xenogeneic bone grafts. However, this method has several limitations in clinical practice, such as the lack of autologous bone and immune responses leading to rejection of allogeneic or xenogeneic bone grafts [4]. The emergence of bone tissue engineering technology has provided a promising new option to solve this problem [5,6].

Bone tissue engineering classically consists of three elements: scaffold, cells with osteogenic potential, and osteogenic-inducing biological factors [7]. Adipose-derived stem cells (ADSC) are considered one of the most ideal cell sources for bone tissue engineering due to the advantages of wide availability, abundant supply, accessible collection, minimal donor side effects, and patient acceptance [8]. Previous studies have demonstrated excellent osteogenic differentiation potential of ADSC both in vitro and in vivo [9,10].

In vitro cell cultures are usually preferred because a sufficient number of cells is required before transplantation [11]. Fetal bovine serum (FBS) has been traditionally used as a supplement for in vitro cell cultures since FBS contains multiple biological factors for cell growth and adhesion. However, considering the clinical usage of cells, using xenogeneic supplements including FBS should be avoided, as they may carry viruses or pathogens [12,13,14]. Studies have proposed xeno-free cell cultures replacing xenogeneic FBS with homologous or autologous serum and platelet-derived supplements [15,16,17]. Research shows that ADSC cultured under xeno-free conditions maintain the characteristics of stem cells, such as multilineage differentiation potential, immunophenotype, and proliferative capacity [18,19].

Scaffold, another essential element in bone tissue engineering, can provide a favorable microenvironment for attachment, proliferation, and osteogenic differentiation of cells [20,21]. Various structures of scaffolds, such as porous structures of sponges, foams, and non-woven fabrics and dense structures of membranes, microspheres, and conduits, have been reported and used for bone tissue engineering [22,23,24,25]. Micro-sized scaffolds have been used as components of 3-dimensional cell aggregates, which play a crucial role in the substrate for cell adhesion and the pathway for cell supply of nutrients and oxygen and waste removal [26]. Gelatin hydrogel microspheres [27,28] and poly (lactic acid) microfiber [29] have been applied for this purpose [30,31,32]. We focused on micropieces of recombinant peptide with human type I collagen α-1 sequence, called Cellnest^TM^, developed by the Fujifilm group [33]. Cellnest^TM^ can create 3-dimensional cell aggregates with a mosaic structure called CellSaic. Reports showed that cells in CellSaic had high viability due to the petal-like shape of Cellnest^TM^, with a high surface area for cell adhesion that played a crucial role in circulating nutrients and waste through the gaps in CellSaic. We also demonstrated the feasibility of CellSaic with stem cells in bone regeneration [34,35,36]. Moreover, one of the advantages over other micro-sized scaffolds is that Cellnest^TM^ is produced by the yeast *Pichia pastoris*, which ensures a non-xenogeneic environment without the risk of infection [37,38]. This advantage implies the possibility of complete xeno-free culture systems by combining Cellnest^TM^ with cells cultured under xeno-free conditions.

The present study investigates the possibility of complete xeno-free culture systems for rat ADSC (rADSC) using xeno-free culture methods in combination with xeno-free Cellnest^TM^ to prepare rADSC-CellSaic. The in vitro biological properties of rADSC or rADSC-CellSaic under xeno-free conditions are compared to those under conventional xenogeneic conditions using an FBS-containing medium. We also compare the in vivo bone regeneration potential using the rat model of mandibular congenital bone defects established by Yaguu et al. [39].

## 2. Results

### 2.1. Characteristics of rADSC under 2-Dimensional Culture by Medium Supplemented with or without Xeno Components

**Figure 1:**
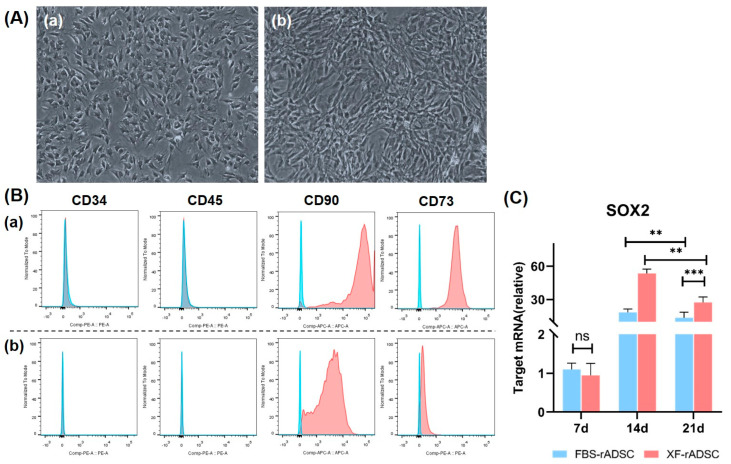
Characteristics of rADSC under two-dimensional culture by medium supplemented with or without xenogeneic components. (**A**) Representative phase contrast microscopic images of FBS-rADSC (**a**) and XF-rADSC (**b**). rADSC with passage 3 and 80% confluency were used. Scale bar: 100 µm. (**B**) Surface antigens expressed on FBS-rADSC (**a**) and XF-rADSC (**b**) using flow cytometric analysis. rADSC with passage 3 and 80% confluency were used. The histograms colored in blue and red indicate the isotype control and experimental group, respectively. (**C**) Time profile of SOX2 gene expression level for FBS-rADSC and XF-rADSC using the RT-qPCR analysis. ** *p* < 0.01, *** *p* < 0.001, ns: no significance.

To explore the possibility of xeno-free culture systems, isolated rADSC were cultured in 2-dimensional dishes with a medium containing FBS of a xenogeneic component (FBS-rADSC) or a xeno-free medium (XF-rADSC), and their morphological and biological characteristics were evaluated (Figure 1). Both FBS-rADSC and XF-rADSC displayed a spindle-shaped fibrous morphology (Figure 1A). Flow cytometric analysis revealed that both FBS-rADSC and XF-rADSC positively expressed CD73 and CD90, typical surface antigens of mesenchymal stem cells, while negatively expressing CD34 and CD45, typical surface antigens of hematopoietic stem cells (Figure 1B). For assessing the stemness of FBS-rADSC and XF-rADSC, the gene expression profile of sex-determining region Y-box transcription factor 2 (SOX2) was evaluated using real-time quantitative polymerase chain reaction (RT-qPCR) (Figure 1C). Both FBS-rADSC and XF-rADSC expressed the SOX2 gene, while gene expression of XF-rADSC was significantly higher than that of FBS-rADSC at 14 and 21 days after culturing.

### 2.2. Formation and Evaluation of 3-Dimensional Aggregates of rADSC and Cellnest (rADSC-CellSaic)

**Figure 2:**
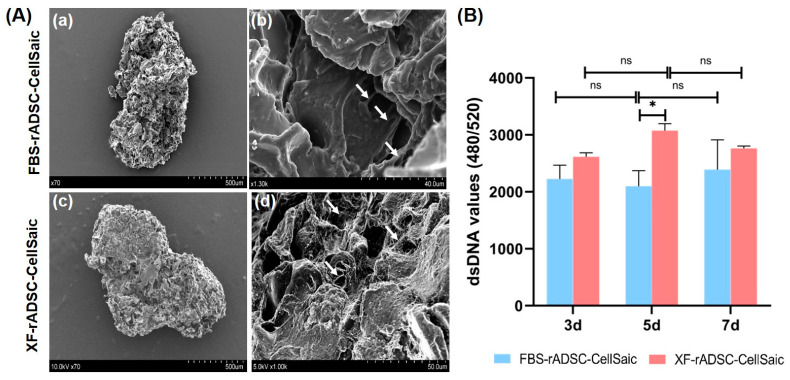
(**A**) SEM images of FBS-rADSC-CellSaic (**a**,**b**) and XF-ADSC-CellSaic (**c**,**d**); whole (**a**,**c**) and enlarged images (**b**,**d**). Arrows indicate cells attached to Cellnest. (**B**) Change of cell number in each CellSaic evaluated by PicoGreen^TM^ assay. * *p* < 0.05, ns: no significance.

FBS-rADSC or XF-rADSC were mixed with xeno-free recombinant peptide micropieces (Cellnest) to form 3-dimensional aggregates of rADSC, called FBS-rADSC-CellSaic or XF-rADSC-CellSaic. Observation by scanning electron microscope (SEM) demonstrated no morphological difference in aggregate structure between FBS-rADSC and XF-rADSC, and both FBS-rADSC and XF-rADSC were stably attached to Cellnest (Figure 2A). There was also no difference in change of cell number between FBS-rADSC-CellSaic and XF-rADSC-CellSaic (Figure 2B), except for 5 days incubation.

**Figure 3:**
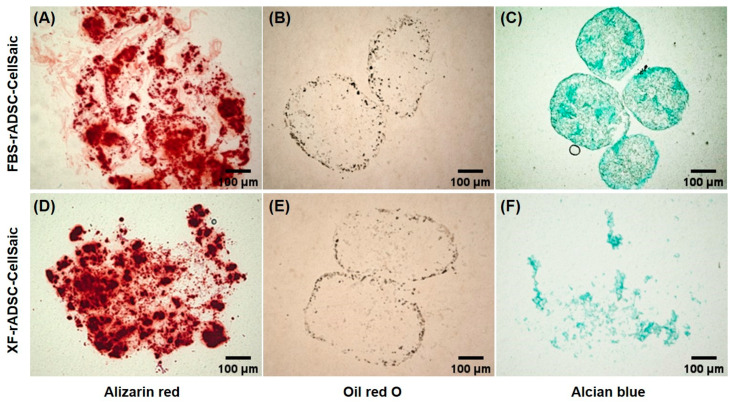
Staining images of FBS-rADSC-CellSaic and XF-rADSC-CellSaic 21 days after differentiation induction. Alizarin red (**A**,**D**), oil red (**B**,**E**), and Alcian blue staining methods (**C**,**F**) were used to evaluate the ability of osteogenic, adipogenic, and chondrogenic differentiation, respectively. Scale bar: 100 µm.

To investigate the multiple differentiation potential into osteogenic, adipogenic, and chondrogenic lineages, FBS-rADSC-CellSaic and XF-ADSC-CellSaic were cultured in each differentiation medium for 21 days, and the extent of differentiation was evaluated by each staining method (Figure 3). Both FBS-rADSC and XF-rADSC in CellSaic were stained by Alizarin red, Oil red O, and Alcian blue. XF-rADSC-CellSaic after the culture with chondrogenic differentiation medium was disintegrated after Alcian Blue staining. These results confirmed that FBS-rADSC and XF-ADSC in CellSaic exhibited multiple differentiation potentials into three lineages.

**Figure 4:**
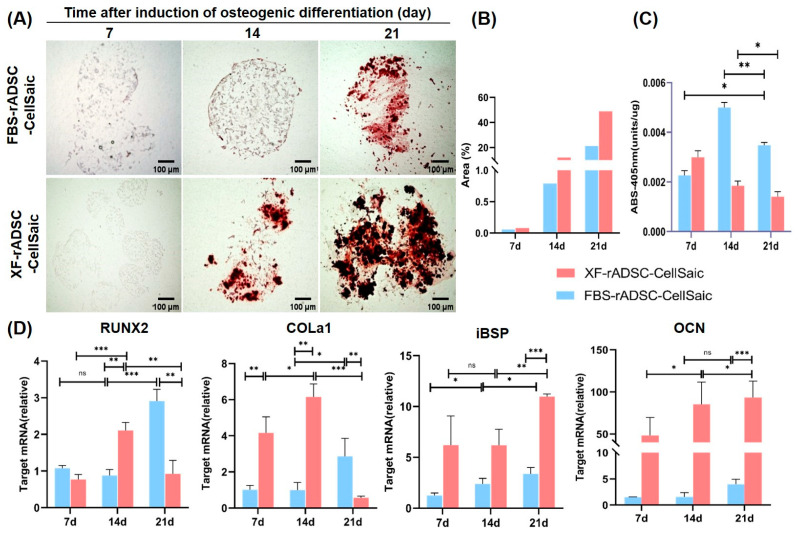
Evaluation of osteogenic differentiation profiles for FBS-rADSC-CellSaic and XF-rADSC-CellSaic. (**A**) Alizarin red staining, (**B**) Percentage of staining area analyzed by Image J version 1.54g. (**C**) ALP activity, and (**D**) Gene expression were evaluated 7, 14, and 21 days after induction of osteogenic differentiation. * *p* < 0.05, ** *p* < 0.01, *** *p* < 0.001, ns: no significance.

The behavior of osteogenic differentiation for FBS-rADSC-CellSaic and XF-rADSC-CellSaic was evaluated in detail by several methods (Figure 4). Little calcium deposition was observed by Alizarin red staining in FBS-rADSC-CellSaic until 14 days after induction of osteogenic differentiation. However, calcium deposition was clearly observed for XF-rADSC-CellSaic after 14 days of differentiation induction (Figure 4A,B). When measured, alkaline phosphatase (ALP) activity for FBS-rADSC-CellSaic reached a maximum at day 7; ALP activity for XF-rADSC-CellSaic decreased with time (Figure 4C). The expression level of genes related to osteogenic differentiation was also evaluated (Figure 4D). For runt-related transcription factor 2 (RUNX2) and type I collagen (COLa1) genes expressed at the initial phase of osteogenic differentiation, the expression levels of FBS-rADSC-CellSaic increased with time while those of XF-rADSC-CellSaic reached a maximum at day 14 and then decreased. For integrin-binding sialoprotein (iBSP) and osteocalcin (OCN) genes expressed at the latter phase of osteogenic differentiation, the expression increased with time for both CellSaics, while FBS-rADSC-CellSaic level was significantly lower than that of XF-rADSC-CellSaic.

### 2.3. Bone Formation in Rat Mandibular Congenital Bone Defects by the Transplantation of rADSC-CellSaic after Osteogenic Differentiation

**Figure 5:**
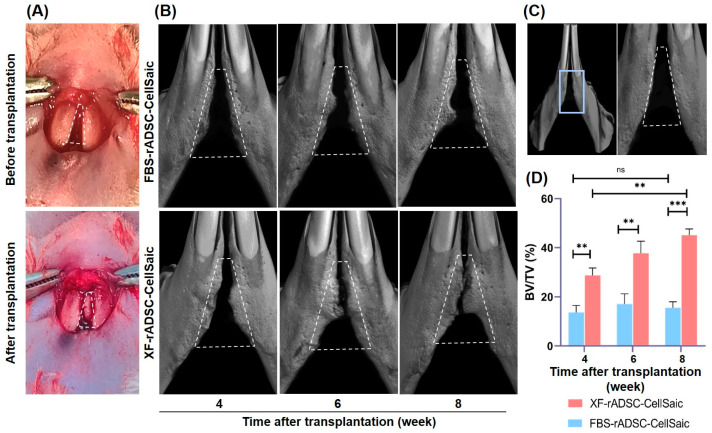
Morphological evaluation of bone formation in rat mandibular congenital bone defect by the implantation of FBS-rADSC-CellSaic or XF-rADSC-CellSaic. (**A**) Representative photographs of bone defect before and after transplantation. (**B**) μCT images of bone defect region 4, 6, and 8 weeks after implantation. (**C**) μCT image of bone defect region 4 weeks after sham operation. (**A**–**C**) Trapezoids with white dashed line indicate the place of initial defect. The regions inside the trapezoids indicate the regenerated new bone. (**D**) Change of BV/TV percentages 4, 6, and 8 weeks after implantation. ** *p* < 0.01, *** *p* < 0.001, ns: no significance.

To investigate the bone formation potential of rADSC-CellSaic after osteogenic differentiation, FBS-rADSC-CellSaic or XF-rADSC-CellSaic were osteogenically differentiated for 21 days and transplanted to mandibular congenital bone defects in rats. Bone formation was observed for both rADSC-CellSaics, while the percentage of bone volume to total volume (BV/TV) for XF-rADSC-CellSaic was significantly higher than that for FBS-rADSC-CellSaic (Figure 5).

**Figure 6:**
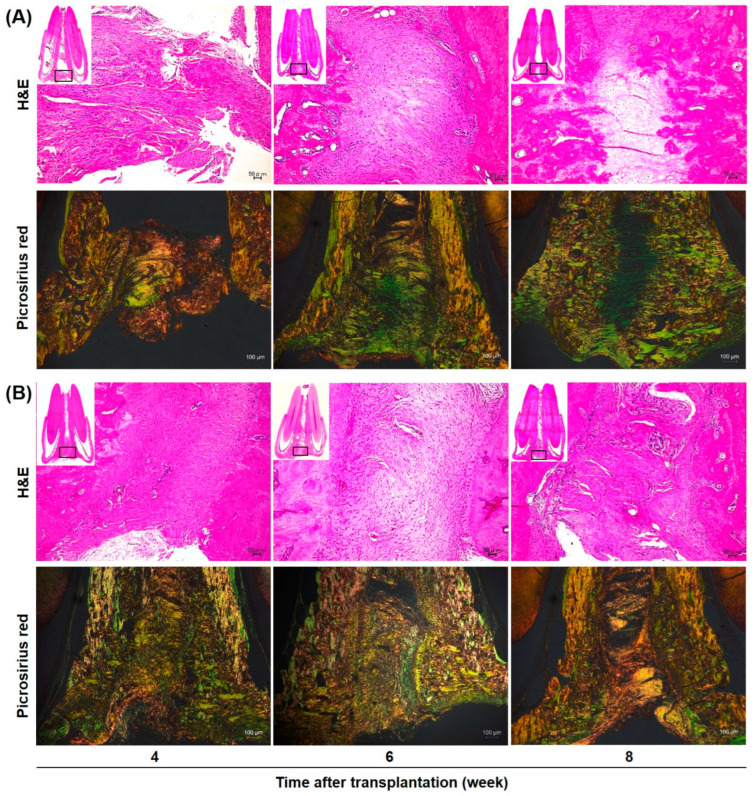
Histological images of bone tissue regenerated by the transplantation of FBS-rADSC-CellSaic (**A**) or XF-rADSC-CellSaic (**B**). HE (upper) or Picrosirius red staining (lower) was performed 4, 6, and 8 weeks after transplantation. Rectangle in a small inset image of HE indicates the region of the enlarged image. Scale bar: 50 µm (HE staining), 100 µm (Picrosirius red staining).

Histological evaluation (Hematoxylin and Eosin (H and E) staining and Picrosirius red staining) was performed 4, 6, and 8 weeks after transplantation of FBS-rADSC-CellSaic or XF-rADSC-CellSaic (Figure 6). H and E staining revealed no infection and inflammation signs for either XF-rADSC-CellSaic or FBS-rADSC-CellSaic. In the FBS-rADSC-CellSaic group, fibrous tissue was observed at the transplant site, but no bone-like tissue was observed even 8 weeks after transplantation. However, in the XF-rADSC-CellSaic group, dense fibrous tissue was observed 4 weeks after transplantation, with partial vascular structure and bone tissue observed at 6 and 8 weeks. Polarization microscopic observation of Picrosirius red staining by polarized light microscopy revealed mainly thin type III collagen fibers (green) in the tissue transplanted with FBS-rADSC-CellSaic and thick type I collagen fibers (orange) in the tissue transplanted with XF-rADSC-CellSaic.

## 3. Discussion

The present study used a novel 3-dimensional construct composed of rADSC and recombinant peptide micropieces (Cellnest) under xeno-free conditions (XF-rADSC-CellSaic) and compared the multilineage differentiation potential in vitro and bone regeneration in vivo to the construct prepared under conventional conditions containing xenogeneic components (FBS-rADSC-CellSaic). Compared to FBS-rADSC-CellSaic, XF-rADSC-CellSaic accelerated and enhanced osteogenic differentiation in vitro and promoted bone regeneration in vivo for mandibular congenital bone defects in rats. Many researchers have demonstrated the importance of stem cell preparation under xeno-free conditions [8,40,41,42] and a 3-dimensional construction of stem cells [43,44] to realize safe and effective regenerative therapy. This report is the first to evaluate the feasibility of 3-dimensional stem cell construction created under complete xeno-free conditions.

At first, the study explored the effect of xeno-free culture on the biological characteristics of rADSC. Evaluation of morphology and representative surface antigens for mesenchymal stem cells (MSC) demonstrated that rADSC maintained the MSC characters even after culturing under xeno-free conditions (Figure 1A,B). These results correspond to the results previously reported [41,45]. Notably, the SOX2 expression level of a stemness-related gene [46] for XF-rADSC was significantly higher than that of FBS-rADSC (Figure 1C). However, the mechanism of stemness maintenance by the xeno-free culture lacks elucidation.

Three-dimensional construction of rADSC under xeno-free conditions (XF-rADSC-CellSaic) was achieved by impregnating xeno-free recombinant peptide micropieces (Cellnest). Generally, cell viability in the interior of large 3-dimensional constructs decreases due to the lack of nutrient and oxygen supply and waste removal [47]. Incorporating Cellnest forms channels for nutrient and waste exchange, contributing to maintaining the viability of cells present even in large 3-dimensional constructs [33]. In the present study, FBS-rADSC and XF-rADSC numbers in CellSaic were maintained for 7 days (Figure 2B). Moreover, both FBS-rADSC and XF-rADSC in CellSaic maintained multilineage differentiation potential (Figure 3). These results strongly indicate that the incorporation of Cellnest is effective in creating large 3-dimensional constructs of stem cells with preserved biological functions. However, XF-rADSC-CellSaic showed a fragile structure after induction of chondrogenic differentiation, probably due to the lack of extracellular matrix deposition during chondrogenic differentiation under xeno-free conditions. More clarity on the molecules involved in efficiently depositing extracellular matrix in chondrogenic differentiation will help solve this issue.

Alizarin red staining, ALP activity, and gene expression during osteogenic differentiation demonstrated that XF-rADSC-CellSaic accelerated and promoted osteogenic differentiation more than FBS-rADSC-CellSaic (Figure 4). One of the conceivable reasons for the higher differentiation capacity of XF-rADSC-CellSaic is the stemness of rADSC in CellSaic. As shown in Figure 1C, the stemness of XF-rADSC was higher than that of FBS-rADSC. The high stemness characteristic of XF-rADSC may be maintained even after the preparation of CellSaic due to the incorporation of Cellnest, resulting in the promotion of osteogenic differentiation.

Micro CT analysis of regenerated bone tissue revealed that the bone-forming potential of XF-rADSC-CellSaic was higher than that of FBS-rADSC-CellSaic (Figure 5). Since another research paper demonstrated the poor potential of regeneration with Cellnest itself [48], the effect of CellSaic without cells was not investigated in this paper. Considering that XF-rADSC-CellSaic exhibited a higher extent of osteogenic differentiation than FBS-rADSC-CellSaic (Figure 4), the superior bone formation by the transplantation of XF-rADSC-CellSaic after osteogenic differentiation indicates a high bone regeneration potential of XF-rADSC-CellSaic remaining even after transplantation. In addition, the histological evaluation (Picrosirius red staining observed by polarized microscopy) revealed the maturation of collagen structure in the new bone tissue transplanted by XF-rADSC-Cellsaic (Figure 6). Considering the importance of collagen structure in bone regeneration [49], these results indicate that transplantation of XF-rADSC-Cellsaic induced bone regeneration with high quality.

There are several limitations to the present study. First, the reason why the stemness of rADSC under xeno-free culture is maintained is still unknown. The mechanism of stemness maintenance by xeno-free cultures needs elucidation at the molecular level in future investigations. Next, in the present study, XF-rADSC-CellSaic, after osteogenic differentiation culture for 21 days, was transplanted to induce bone regeneration. Other conditions, such as the number of rADSC-CellSaics for transplantation and the transplantation of rADSC-CellSaics without osteogenic differentiation in advance, should be investigated not only to optimize bone regeneration but also to evaluate the bone regeneration potential of undifferentiated rADSC combined with Cellnest of a scaffold.

## 4. Materials and Methods

### 4.1. Isolation of rADSC

In this study, animal experiments, including rADSC isolation and transplantation of rADSC-CellSaic into rat mandibular congenital bone defects (Section 4.4), were performed. These animal experiments were approved by the local ethics committee of Osaka Dental University (Approval number: 2301001) and strictly performed according to the guidelines. The isolation of rADSC was performed as previously reported [10]. Briefly, the adipose tissue was collected from the fat pad in the inguinal region of 6-week-old male F344 rats (3 rats) (SHIMIZU Laboratory Supplies Co., Ltd., Kyoto, Japan). Then, the adipose tissue was minced and immediately digested using phosphate-buffered saline (PBS) containing type I collagenase (FUJIFILM Wako Co., Osaka, Japan) (0.3 wt%) at 37 °C for 45 min, stirring the mixture every 15 min. After digestion, the tissue was centrifuged and filtered with a mesh (100 μm) to separate the cells from the surrounding tissue. The obtained cells were seeded on a cell culture dish and designated as passage 0. After culturing the cells under standard conditions (37 °C, 5% carbon dioxide) for 24 h, the dish was washed twice with PBS to remove any non-adherent cells. Only the adherent cells were regarded as rADSC. The culture medium was changed every 3 days. The cells were expanded in a T75 flask (IWAKI Cell Biology, AGC Inc., Tokyo, Japan) and passaged on reaching 80% confluency.

### 4.2. Two-Dimensional Culture of rADSC by the Medium with or without Xenogeneic Components

This study used two types of media for culturing rADSC: the usual medium and the xeno-free medium. Dulbecco modified Eagle medium (DMEM) with low glucose (FUJIFILM Wako Co., Osaka, Japan) supplemented with 1% antibiotics (Thermo Fischer Scientific Inc., Carlsbad, CA, USA) and 10% fetal bovine serum (FBS, Hyclone, Cytiva., Logan, UT, USA) was used as the usual medium. The rADSC cultured with the medium containing FBS (FBS-rADSC) were passaged using 1% trypsin-ethylenediaminetetraacetic acid (Lonza, Biowhittaker, Belgium). However, Cellartis^®^ MSC Xeno-Free Culture Medium (Takara Bio Inc., Kusatsu, Japan) and culture dish pre-coated with iMatrix-511(Nippi Inc., Tokyo, Japan) were used for a xeno-free culture of rADSC. The rADSC cultured with xeno-free medium (XF-rADSC) were passaged using TrypLE Select (Thermo Fischer Scientific Inc., Carlsbad, CA, USA) [19]. The rADSC with the passage number of 3 were used in the following experiments.

Surface antigens expressed on FBS-rADSC or XF-rADSC were analyzed using flow cytometry. Antibodies used for staining surface antigens were anti-CD73 antibody conjugated with Phycoerythrin (CD73-PE, Bioss Antibodies Inc., Boston, MA, USA), anti-CD90 antibody conjugated with Allophycocyanin (CD90-APC, BioLegend Inc., San Diego, CA, USA), anti-CD45 antibody conjugated with PE (CD45-PE, BioLegend Inc., San Diego, CA, USA), and anti-CD34 antibody conjugated with PE (CD34-PE, Novus Bio-logicals, LLC, USA). Mouse immunoglobulin G (IgG) 2a and IgG1 (Thermo Fisher Scientific Inc., Waltham, MA, USA) were used as isotype controls. The rADSC with passage number 3 were resuspended with PBS containing 2% FBS and incubated with each antibody at 4 °C for 30 min to stain corresponding surface antigens. The suspension of antibody-stained cells was analyzed using FACSVerse (BD Biosciences, Franklin Lakes, NJ, USA), and the collected data were further analyzed using FlowJo X software version 10.8.0 (TreeStar Inc., Ashland, OR, USA).

The profile of stemness-related gene expression for FBS-rADSC or XF-rADSC was evaluated using the RT-qPCR method. Total RNA of rADSC cultured with growth medium for 7, 14, and 21 days was extracted using the RNeasy Mini Kit (QIAGEN, Hilden, Germany) according to the manufacturer’s instructions. Then, the reverse transcription of extracted total RNA was used to synthesize complementary DNA (cDNA) using SuperScript™ IV VILO™ Master Mix (Thermo Fisher Scientific Inc., Waltham, MA, USA). After mixing cDNA, TaqMan^TM^ Fast Advanced Master Mix (Thermo Fisher Scientific Inc., Waltham, MA, USA), RNase-free water, and TaqMan^TM^ Gene Expression Assays (Thermo Fisher Scientific Inc., Waltham, MA, USA) for SOX2 (Rn01286286_g1) or glyceraldehyde-3-phosphate dehydrogenase (rat GAPDH endogenous control; Thermo Fisher Scientific Inc., Waltham, MA, USA), the RT-qPCR was carried out using the One Step Plus PCR system (Thermo Fisher Scientific Inc., Waltham, MA, USA). The reaction condition was as follows: enzyme activation; 50 °C for 2 min, denaturation; 95 °C for 20 s, holding 95 °C for 1 s, annealing; 60 °C for 20 s, PCR cycles; 40. Data analysis was conducted using the ΔΔ Ct method, while GAPDH was selected as an internal reference. The experiment was repeated 3 times.

### 4.3. Preparation and Evaluation of rADSC-CellSaic In Vitro

An equal volume (200 μL) of rADSC (1.0 × 10^5^ cells/mL) and Cellnest™ (0.1 mg/mL, FUJIFILM CO., Minato, Tokyo, Japan) suspended in the basal culture medium was added to PrimeSurface 96U plate (Sumitomo Bakelite Co., Ltd., Tokyo, Japan). The plate was centrifuged (600× *g*, 5 min) and incubated for 24 h to obtain a 3-dimensional cell aggregate (rADSC-CellSaic). A larger cell aggregate formed by culturing 8 pieces of rADSC-CellSaic in PrimeSurface 96U plate for 48 h was used for following in vitro evaluations. Medium of rADSC-CellSaic was changed every 3 days.

The rADSC-CellSaic cultured for 21 days was fixed with 4% paraformaldehyde and dehydrated. After critical point drying using a vacuum device (HCP-2, Hitachi High-Tech., Co., Tokyo, Japan), the sample was coated with osmium using the HPC-20 osmium coater (Vacuum Device Co., Ltd., Ibaraki, Japan), followed by observation using a scanning electron microscope (FE-SEM; S-4800; Hitachi; Tokyo, Japan) at an accelerating voltage of 5 kV or 10 kV.

The number of rADSC in CellSaic cultured for 3, 5, and 7 days was measured using the Quat-iT^TM^ PicoGreen^TM^ dsDNA Kit (Thermo Fisher Scientific Inc., Waltham, MA, USA) under the manufacturer’s instructions. The fluorescent intensity of the mixture was measured with SpectraMax^TM^ M5e (Molecular Devices, LLC., Tokyo, Japan) at the excitation and emission wavelengths of 480 and 520 nm, respectively.

The multilineage (osteogenic, adipogenic, and chondrogenic) differentiation of rADSC in CellSaic was induced by culturing for 21 days with growth medium containing 50 μM ascorbic acid (FUJIFILM Wako Co., Osaka, Japan), 10 mM beta-glycerophosphate (FU-JIFILM Wako Co., Osaka, Japan), and 0.1 μM dexamethasone (FUJIFILM Wako Co., Osaka, Japan), MSC-ADDM differentiation medium (Cosmo Bio Co., Ltd., Tokyo, Japan), and PRIME-XV chondrogenic differentiation medium (Irvine Scientific, Santa Ana, CA, USA). During differentiation culture, the medium was gently replaced every 3 days. The rADSC-CellSaic, after each differentiation induction, was washed with PBS and fixed with 4% paraformaldehyde, followed by the preparation of frozen sections with 5 µm thickness. The section was stained with Alizarin red S (Sigma-Aldrich, St. Louis, MO, USA), Oil Red O (FUJIFILM Wako Co., Osaka, Japan), and Alcian blue (Sigma-Aldrich, St. Louis, MO, USA) to evaluate the behavior of osteogenic, adipogenic, and chondrogenic differentiation, respectively. The stained sections were observed under a digital microscope (BZ-9000; Keyence Co., Osaka, Japan).

For osteogenic differentiation, the rADSC-CellSaics of differentiation culture for 7, 14, and 21 days were collected and evaluated by several methods. First, calcium deposition was evaluated by Alizarin red staining, in which the procedure was the same as described above. The Image J version 1.54g (National Institutes of Health, Bethesda, MD, USA) was used for the semi-quantitative analysis of staining results, which illustrate the percentage of staining area. Second, the rADSC-CellSaic was lysed with a matrix lysis solution, and the DNA amount in the lysate was measured using the PicoGreen^TM^ DNA quantification kit (Thermo Fisher Scientific Inc., Waltham, MA, USA). The ALP activity was measured using the LabAssay^TM^ ALP kit (FUJIFILM CO., Tokyo, Japan) and normalized based on the cellular DNA content. The expression level of osteogenic genes (RUNX2, COLa1, iBSP, and OCN) was evaluated using the RT-qPCR method. The procedure was the same as described above. The accession numbers of the TaqMan^TM^ Gene Expression Assay used for PCR are as follows: RUNX2, Rn01512298_m1; COL1A1, Rn01463848_m1; BSP, Rn00561414_m1; GAPDH, Rn01775763_g1 OCN, Rn00566386_g1. These experiments were repeated three times.

### 4.4. Transplantation of rADSC-CellSaic after Osteogenic Differentiation into Rat Mandibular Congenital Bone Defect

Rat mandibular congenital bone defects were created based on the procedures reported by Sasayama et al. [36]. Male 8-week-old F344 rats were anesthetized by intraperitoneal injections of a mixture solution composed of medetomidine hydrochloride (0.15 mg/kg; Zenoaq, Fukushima, Japan), midazolam (2 mg/kg; Sandoz KK, Yamagata, Japan), and butorphanol tartrate (2.5 mg/kg; Meiji Seika Pharma Co., Ltd., Tokyo, Japan). In addition, the rat mandibular cleft received local anesthesia with lidocaine (SHOWA YAKUHIN KAKO CO., LTD., Japan). The skin at the inferior margin of the mandible was incised, and the defect space was exposed by detaching the soft tissue and mandibular symphysis. Then, 32 pieces of rADSC-CellSaic were transplanted into the defect space. After transplantation, the skin and mucous membrane above the transplanted area were separately sutured. In this animal experiment, 24 rats were used and divided into two groups (12 rats each): transplantation of FBS-rADSC-CellSaic after osteogenic differentiation for 21 days, and transplantation of XF-rADSC-CellSaic after osteogenic differentiation for 21 days. No transplantation into the defect was used as a negative control. The skin overlying the mandibular cleft of the rats was incised to expose the mandible [39,50].

The tissue surrounding the mandibular clefts was collected 4, 6, and 8 weeks after transplantation and fixed with 4% paraformaldehyde. Radiopacity and morphology of bone newly formed at the defect site were evaluated using µCT scans (Bruker Skyscan 1172, Bruker, Kontich, Belgium) with a 0.5 mm aluminum filter and a 100 μA current at 50 kV radiation. A 3-dimensional image of the mandible was reconstructed using CTAN image analysis software version 1.17.7.2+ (Bruker, Kontich, Belgium). Cylindrical pieces containing different hydroxyapatite contents ranging from 200 to 1550 mg/cm^3^ were used to evaluate the bone mineral density of calcified bone tissue and measure bone volume density (BV/TV%).

Fixed tissues were decalcified using EDTA (Nicolai Tissue, Inc., Kyoto, Japan) and dehydrated. After embedding the dehydrated samples in paraffin, thin sections (5 µm thickness) were prepared and stained with Hematoxylin-Eosin and Picrosirius red according to the manufacturer’s instructions. All images were captured using a polarized light microscope (ECLIPSE Ci POL, Nikon, Tokyo, Japan).

### 4.5. Statistical Analysis

Statistical analysis was performed using SPSS 22.0 software (IBM Corp., Armonk, NY, USA). Quantification of cell ALP activity, mineral deposition, real-time fluorescence quantitative PCR results, and semi-quantitative analysis of mineralized tissue were analyzed using one-way analysis of variance (ANOVA), with a significance level of *p* < 0.05. For homogeneity of variance, pairwise comparisons were conducted using the Bonferroni method. If the data did not meet the assumption of equal variances, pairwise comparisons were performed using the rank sum test. The significance level was adjusted using the Bonferroni correction, and a value of *p* < 0.01 was considered statistically significant.

## Data Availability

Data are contained within the article.
